# The novel protein KBP regulates mitochondria localization by interaction with a kinesin-like protein

**DOI:** 10.1186/1471-2121-6-35

**Published:** 2005-10-14

**Authors:** Marcin J Wozniak, Martina Melzer, Cornelia Dorner, Hans-Ulrich Haring, Reiner Lammers

**Affiliations:** 1 Medical Clinic IV, Otfried-Müller Str.10, Tübingen, Germany; 2 University of Manchester, Manchester, UK; 3 Boehringer Ingelheim Pharma KG, Biberach an der Riss, Germany

## Abstract

**Background:**

Members of the Kinesin-3 family of kinesin-like proteins mediate transport of axonal vesicles (KIF1A, KIF1Bβ), distribution of mitochondria (KIF1Bα) and anterograde Golgi to ER vesicle transport (KIF1C). Until now, little is known about the regulation of kinesin-like proteins. Several proteins interact with members of this protein family. Here we report on a novel, ***K***IF1 ***b***inding ***p***rotein (KBP) that was identified in yeast two-hybrid screens.

**Results:**

KBP was identified by using the yeast-two-hybrid system with an amino-terminal fragment of KIF1C as a bait that is strongly homologous to KIF1B. Here we investigated the interaction of KBP and KIF1B. The full length proteins coimmunoprecipitated after overexpression and in untransfected 293 cells. Immunofluorescence experiments revealed that KBP was mainly localized to mitochondria, as has been described for KIF1Bα. Overexpression of a deletion mutant or reduction of the KBP protein level using an anti-sense construct led to an aggregation of mitochondria. Such an effect is probably due to the lower activity of KIF1Bα in the absence of KBP, as was revealed in motility assays.

**Conclusion:**

KBP is a new binding partner for KIF1Bα that is a regulator of its transport function and thus represents a new type of kinesin interacting protein.

## Background

Intracellular transport in cells is mediated by three different types of motor proteins that travel along filamentous tracks. Actin filaments are used by the myosin family of proteins, while transport along microtubules (MT) is mediated by either kinesin-like proteins (KLPs) or dyneins. The functional difference between these two kinds of molecular motors lies in the polarity of movement. Dyneins transport their cargoes toward the minus end of MT, while most KLPs transport cargo towards the minus and plus end of MT, depending on the position of the motor domain at the amino- or carboxyl-terminus of the protein (for reviews see [1,2]). The common feature of all KLPs is a high degree of homology in their motor domain, a 340 amino acids region that contains MT- and ATP-binding sites and classifies them into one of 14 families of the kinesin superfamily [3]. Human and mouse genomes encode 45 KLPs [4] whose functions classically are transport of cargo, like protein rafts, lysosomes, chromosomes or various membrane vesicles. However, KLPs also can zipper, cross-link and influence the stability of MT for building and maintaining the mitotic and meiotic spindle apparatus (for reviews see [5-7].

Our previous research identified the protein KIF1C that localizes to the Golgi apparatus. Using a dominant-negative mutant we have shown that KIF1C is involved in transport of vesicles from the Golgi to the ER [8]. Together with KIF1A and KIF1B, KIF1C belongs to the Kinesin-3 family. The three proteins show a high degree of sequence homology outside their amino-terminal motor domain and share a so called U104 domain. The biological function of this domain, also known as forkhead homology-associated domain [9] has not yet been described in KLPs but it may be involved in protein-protein interactions regulated by phosphorylation. Moreover, KIF1C has been implicated in the susceptibility of mouse macrophages to anthrax lethal toxin [10].

The other member of the Kinesin-3 family, KIF1B, is expressed as two main splicing variants that share 660 amino-terminal amino acids and have different roles in intracellular transport. The α-isoform associates with mitochondria [11], while the β-isoforms [12,13]. are involved in transport of synaptic vesicles and lysosomes in non-neuronal cells [14]. A missense mutation in the ATP binding domain of the motor may be causal for the development of Charcot-Marie-Tooth disease type 2A [15].

For the Kinesin-3 KLPs several binding proteins were identified. KIF1A binds to Liprin-α [16], and KIF1Bα interacts with the PDZ domain of the glucose transporter 1 binding protein, indicating a possible additional target of this KLP [17]. In addition, PDZ domains of PSD-90, PSD-97 and S-SCAM are responsible for interaction with KIF1Bα as well [18]. We reported previously on the binding of protein tyrosine phosphatase D1 [8] and 14-3-3 proteins [19] to KIF1C. Here we describe the identification of a novel KIF1Bα interacting protein, KBP (***K***IF1 ***b***inding ***p***rotein). We show that KBP colocalizes with mitochondria and that it interacts with KIF1Bα. Moreover, we present evidence that this new protein plays a role for the regulation of mitochondrial distribution by regulating the KIF1Bα activity.

## Results

### Identification and cloning of KBP

We have previously identified two proteins that bind to KIF1C, PTPD1 and members of the 14-3-3 protein family [8,19]. To search for proteins that bind to KIF1C between the motor domain and the PTPD1 binding region, we employed the yeast two-hybrid system. Two screens were made, first with amino acids 435 – 622 as a bait, to identify possible U104 binding proteins, and second with amino acids 261 – 800 as a bait. Only the second screen with the larger bait resulted in possible interacting proteins. Out of 5 × 10^6 ^screened transformants, 10 independent isolates of the same cDNA were found to interact.

**Figure 1:**
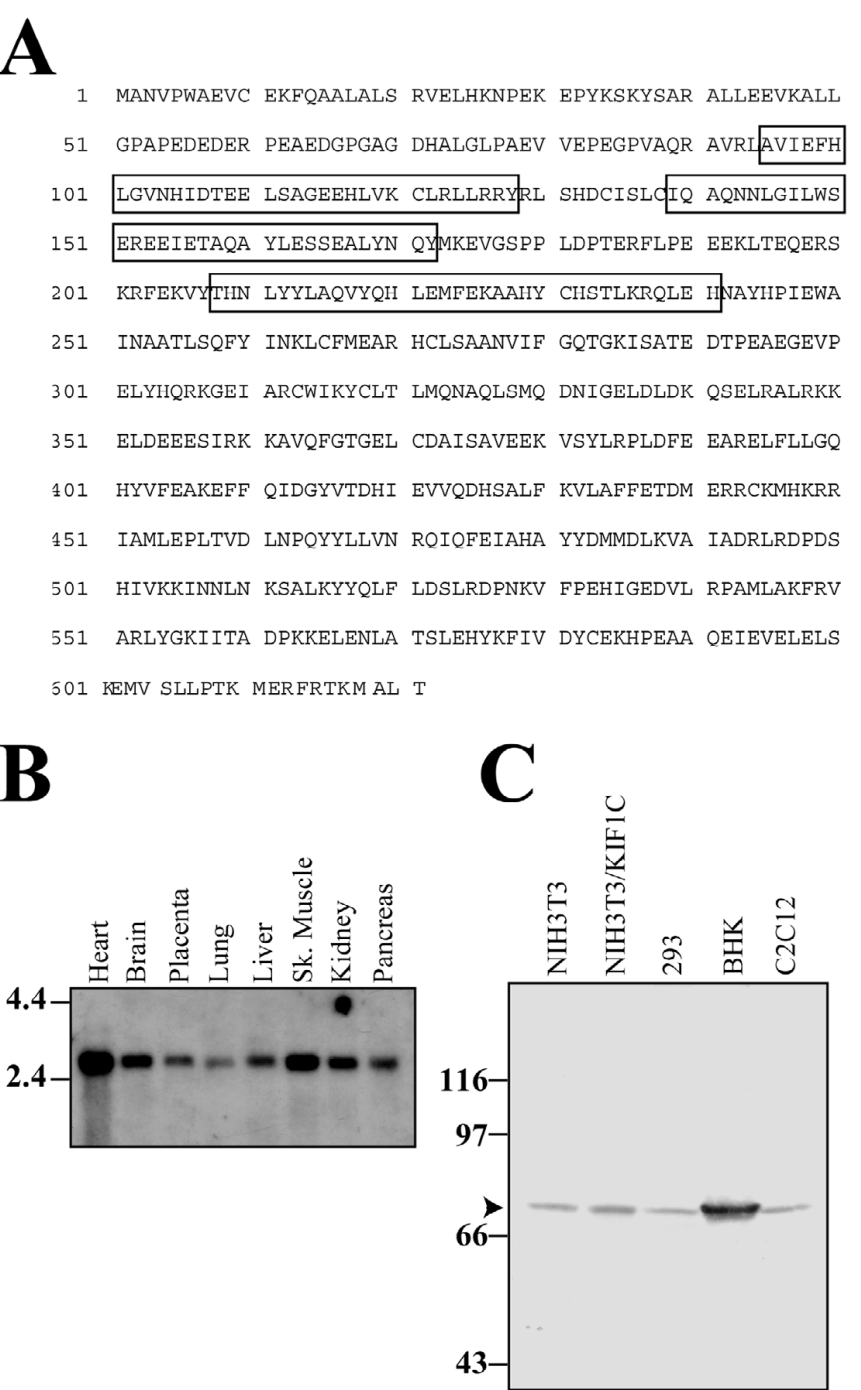
**Expression analysis of KBP**. **A**, amino acid sequence of KBP, probable TPR repeats are boxed. **B**, expression of KBP mRNA in various human tissues was analysed by Northern blot hybridization using the complete cDNA as a probe. The size marker is indicated in kilobase. **C**, expression of KBP in human, mouse and hamster cell lines. Triton cell lysates were separated by SDS-PAGE, transferred to nitrocellulose and immunoblotted using the KBP antiserum. Size markers are indicated in kDa.

The open reading frame of the identified cDNA encodes a protein of 621 amino acids (Fig. 1A), with a calculated molecular mass of 71.8 kDa. A search in the Conserved Domain Database showed weak homology to tetratricopeptide repeat domains (amino acids 95–241) and revealed a very acidic region (amino acids 54–64). The new protein was called KBP (***K***IF1 ***b***inding ***p***rotein). A protein BLAST search revealed that the KBP protein sequence had been identified before and exists in the HUGE Protein Database as KIAA1279.

### Expression of KBP

To estimate the transcript size and prove that the identified clone encodes the complete open reading frame, as well as to check the tissue distribution, Northern blot analysis using the whole cDNA as a probe was performed. A band of 2.6 kb was detected in all examined tissues, with the highest expression found in heart and skeletal muscle (Fig. 1B), which is similar to the expression pattern of KIF1B, as shown by Nangaku et al. [11] and Chen et al. [20]. Next, we constructed a glutathione-S-transferase-KBP (GST-KBP) fusion protein and raised a rabbit polyclonal antiserum against it. When probing lysates from various cell lines with this antibody, a protein of approximately 72 kDa was detected (Fig. 1C). This is in agreement with the predicted molecular mass, and the size of the protein in human cells corresponded with the size of transiently overexpressed protein after transfection, indicating that we indeed cloned the complete open reading frame.

### Localization of KBP in NIH3T3 cells

**Figure 2:**
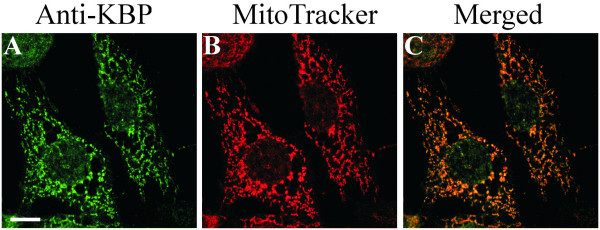
**Intracellular localization of KBP**. The localization of KBP was determined by immunofluorescence analysis in untransfected NIH3T3 (**A**) cells, compared to MitoTracker staining (**B**) and the pictures subsequently merged (**C**). Bars, 10 μm.

To elucidate the function of KBP, we performed immunofluorescence experiments utilising affinity-purified antibodies. Immunostaining of NIH3T3 cells revealed that KBP localized mainly on vesicular structures throughout the cell, which were reminiscent of mitochondria (Fig. 2A). In the same cells, mitochondria were detected with MitoTracker (Fig. 2B), and the merged pictures show that KBP mainly localized to mitochondria (Fig. 2C). These results suggested that the main cellular binding partner for KBP is found at the mitochondria.

**Figure 3:**
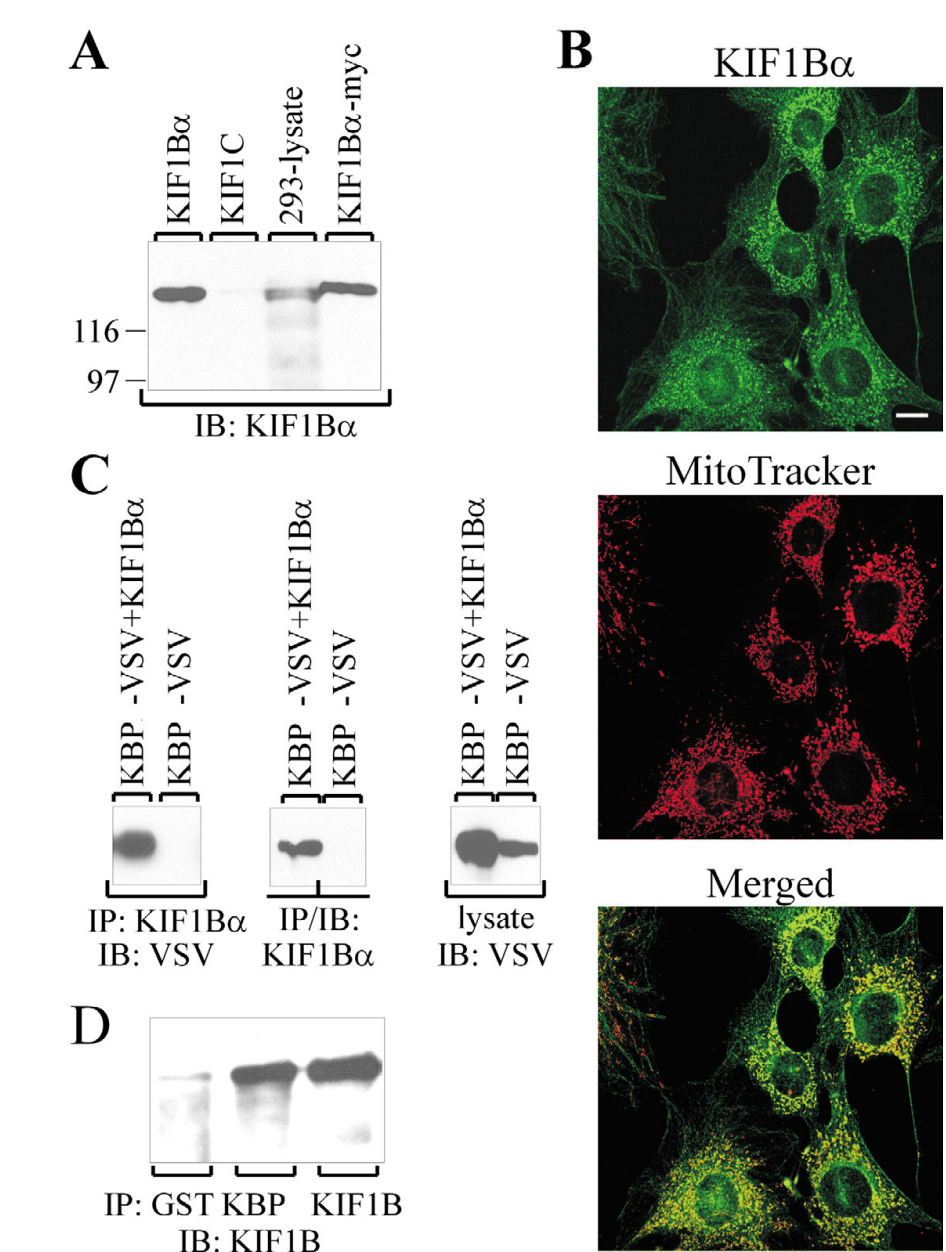
**Expression of human KIF1Bα and interaction with KBP**. **A**, lysates of 293 cells overexpressing KIF1Bα, KIF1Bα-myc and KIF1C or of untransfected 293 cells (10 fold amount of protein) were separated by SDS-PAGE, transferred to nitrocellulose and blotted with antiserum raised against the carboxyl-terminal part of KIF1Bα. **B**, affinity purified antibodies against KIF1Bα (top) and MitoTracker (middle) were used to stain NIH3T3 cells. The merged picture is shown at the bottom. Bar, 20 μm. **C**, KIF1Bα was immunoprecipitated from lysates of 293 cells overexpressing KBP-VSV either alone or together with KIF1Bα. Analysis was done as described and blotting performed with VSV monoclonal antibody. To confirm expression of the proteins, the filter was reprobed with KIF1Bα antibody, while KBP-VSV expression was detected in a separate aliquot of the cell lysates. **D**, untransfected BHK cells were lysed, proteins immunoprecipitated with the indicated antibodies against GST (control), KBP and KIF1Bα and analysed as before by blotting with anti-KIF1B antibody. Molecular mass markers are shown in kDa.

The KLP KIF1Bα is highly homologous to KIF1C, especially within the region used for the yeast two-hybrid screen, and colocalizes with mitochondria [11]. To study the possible interaction between KBP and KIF1Bα we cloned the cDNA of human KIF1Bα and raised antibodies against the carboxyl-terminal part of the protein, a region not present in the KIF1Bβ isoforms. Our antiserum did not cross-react with the homologous KIF1C and recognized an endogenous protein that was of the same size as the cloned one (Fig. 3A). We then tested the antiserum in an immunofluorescence experiment using NIH3T3 cells and, as expected, detected mitochondria-like structures (Fig. 3B). Staining along MTs was also visible (Fig. 3B – top). In a parallel staining, the cells were probed with MitoTracker, and merging of the pictures indicated an identical pattern. This experiment confirmed that we had cloned a human ortholog of the mitochondria binding α-isoform of the KIF1B protein. In addition, the experiment suggested that KBP and KIF1Bα colocalize and that colocalization likely is mediated through a direct interaction of the two proteins.

This interaction of KBP and KIF1Bα was confirmed by co-immunoprecipitation. Transfected 293 cells expressed either a VSV-epitope tagged form of KBP or KBP-VSV plus KIF1Bα. Cells were lysed, the proteins immunoprecipitated using anti-KIF1B serum, separated by SDS-PAGE, transferred to nitrocellulose and blotted with VSV antibody. As shown in Fig. 3C, KBP co-immunoprecipitated with KIF1B when both proteins were overexpressed. Finally, we tested the interaction of KBP and KIF1B in untransfected BHK cells (Fig. 3D). After immunoprecipitation of KBP, KIF1B was detected as a co-immunoprecipitated protein on a Western blot. In conclusion, KBP interacts and colocalizes with KIF1Bα.

### Deregulation of KBP leads to aggregation of mitochondria

**Figure 4:**
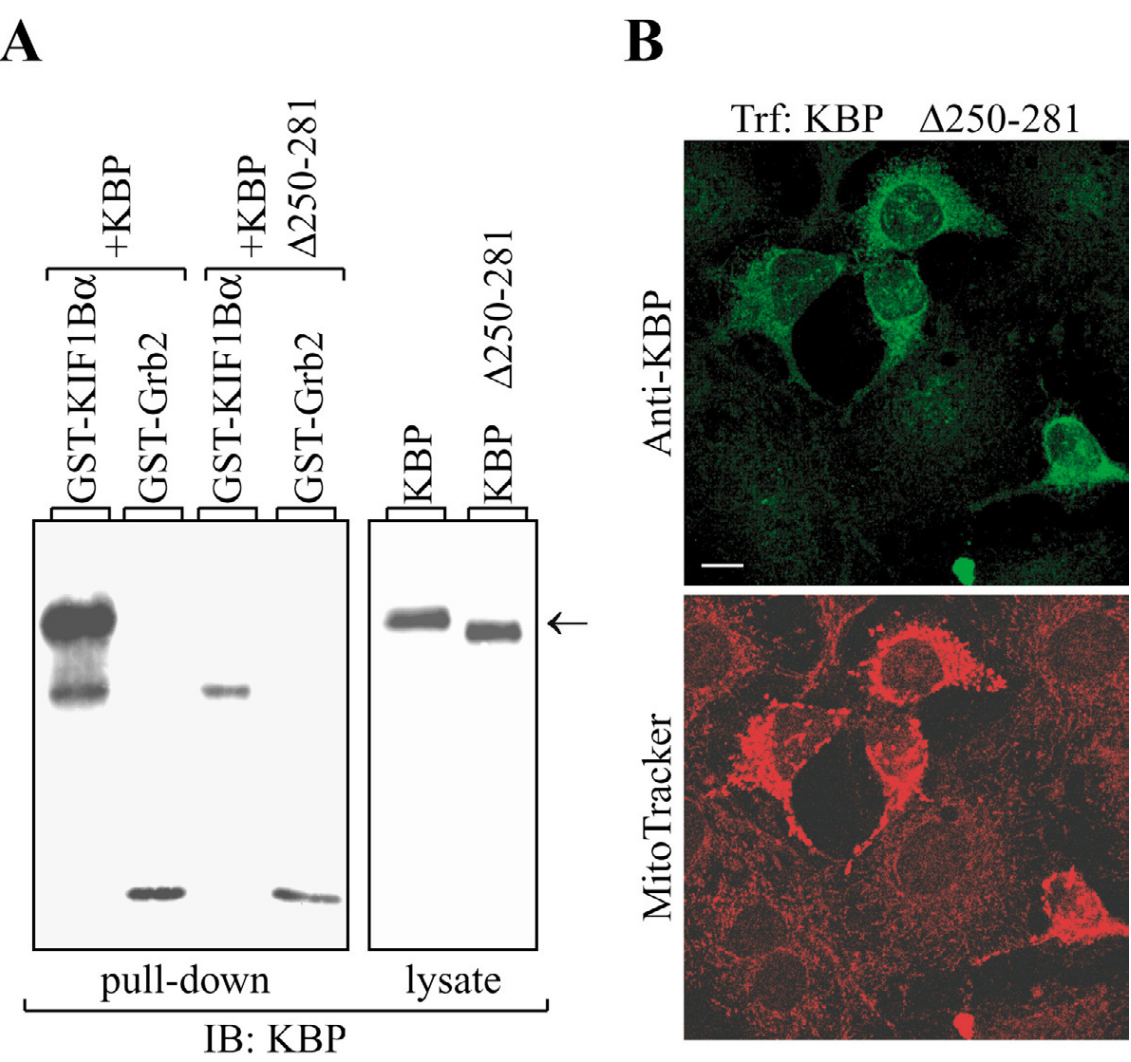
**Expression of KBPΔ250–281 leads to aggregation of mitochondria**. **A**, 1 μg of GST-KIF1Bα fusion protein was incubated with equal amounts of lysates from either KBP or KBPΔ250–281 expressing 293 cells. As a control, GST-Grb2 fusion protein was used. The proteins were separated by SDS-PAGE, transferred to nitrocellulose and immunoblotted with GST-KBP antiserum (left panel). To confirm expression of KBP and the KBP deletion mutant, an equal amount of each lysate was loaded on the gel and analysed with KBP antiserum (right panel). Molecular mass markers are shown in kDa, and an arrow indicates the position of KBP. **B**, NIH3T3 cells transiently overexpressing the KBPΔ250–281 mutant protein were costained with KBP antibody (upper picture) and MitoTracker (lower picture). Trf, transfection; bar, 10 μm.

KIF1Bα was identified as a motor protein responsible for the intracellular transport of mitochondria [11]. Since KIF1Bα and KBP associated with each other and both proteins localized to mitochondria the question arose whether KBP could be a link between the motor protein and this organelle or whether it could play a role in mitochondria distribution. To investigate this question we used two approaches: overexpression of a deletion mutant of KBP that can not interact any more with KIF1Bα, and reduction of the KBP expression level using an anti-sense construct. For the first approach, we employed a deletion mutant of KBP lacking amino acids 250–281. This region was chosen because experiments using GST-fusion proteins indicated that the first 305 amino acids are sufficient for interaction, whereas a mutant lacking the first 214 amino acids was still able to bind KIF1B (data not shown). KBPΔ250–281 was not able to bind to a GST-KIF1Bα(270–641) fusion protein in a pull-down assay, as shown in Fig. 4A. When the KBP mutant was overexpressed in NIH3T3 cells followed by KBP antibody/MitoTracker staining (Fig. 4B) a mitochondrial aggregation was observed in the transfected cells. Overexpression of intact KBP did not affect mitochondrial localization or the organization of the microtubule network (data not shown).

**Figure 5:**
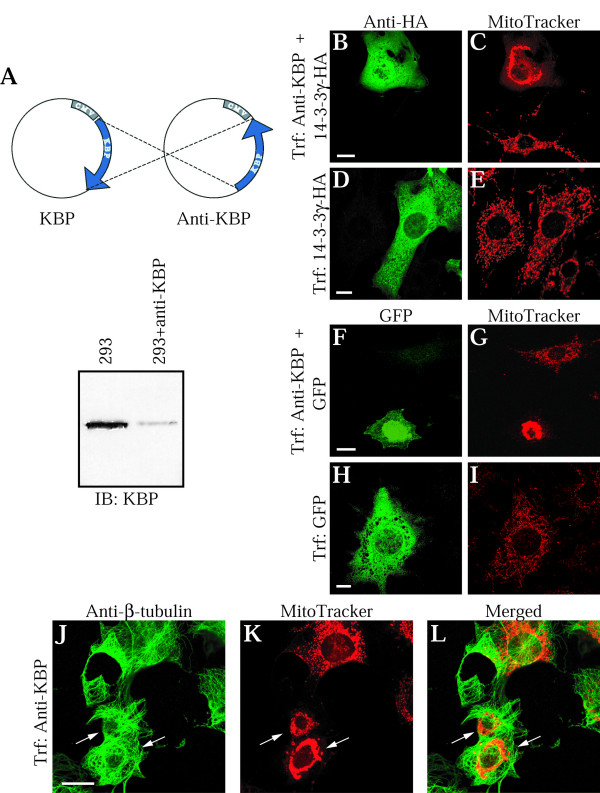
**KBP is essential for a regular distribution of mitochondria in NIH3T3 cells**. **A**, a scheme of the anti-KBP plasmid is depicted. The reduction of endogenous KBP in 293 cells after transient transfection with the anti-KBP plasmid is shown. (**B, C**) NIH3T3 cells were transiently transfected with anti-sense KBP and 14-3-3γ-HA constructs in a 10:1 ratio, respectively, and costained with HA monoclonal antibody (**B**), and MitoTracker (**C**). As a control, NIH3T3 cells were transiently transfected with 14-3-3γ-HA only (**D, E**). Similar as in B-E, cotransfection with GFP was performed (**F-I**). Alternatively, anti-sense KBP transfected cells were stained with antibodies against β-tubulin (**J**) and with MitoTracker (**K**). Arrows in J, K indicate transfected cells. The merged picture of J and K is shown in (**L**). Trf, transfection; bars, 20 μm.

To support these data and to confirm a function of KBP in mitochondrial distribution in an additional and independent approach, we cloned the KBP cDNA in a reversed orientation into the cytomegalovirus promoter based expression vector. Transfection of this construct into 293 cells reduced the expression of endogenous KBP to an overall 30%, when compared to untransfected cells and as detected by blotting analysis (Fig. 5A). Based on FACS-analysis of transfected cells, we assume a transfection efficiency of some 80% with our protocol. This would predict a down-regulation of the KBP protein level to less than 20% in an individual transfected cell. Transfected cells were marked for immunofluorescence inspection by including a plasmid encoding HA-epitope tagged 14-3-3γ in the transfections at a 1:10 ratio. 14-3-3 proteins are very abundant in cells and, as concluded from experiments with 293 cells [19], their expression level is only increased two- to threefold after transfection using optimal amounts of DNA. Therefore, we did not expect an impact of exogenous 14-3-3γ-HA on the mitochondrial distribution process. After transfection, cells were double-stained with monoclonal anti-HA antibodies to identify the transfected cells and with MitoTracker to investigate effects of a reduced KBP expression on mitochondria. In Fig. 5B,C, two cells are in the frame of the picture, and the upper cell has been transfected and therefore stained with the HA-antibody. In C, staining of the mitochondria revealed the regular distribution in the untransfected cell, but an aggregated status in the transfected cell that would contain a reduced amount of KBP. To confirm that this effect was not the result of 14-3-3γ-HA overexpression, NIH3T3 fibroblasts were transfected with the 14-3-3γ-HA expression plasmid alone and anti-HA antibody/MitoTracker staining was performed. Fig. 5D,E show that overexpression of 14-3-3γ-HA did not cause the mitochondria to aggregate. In addition, when similar experiments were conducted using overexpression of GFP similar results were obtained (Fig. 5F–I). To exclude the possibility that mitochondrial aggregation is a result of MT depolymerization or changes in the cell shape, the NIH3T3 cells were transfected with the anti-sense KBP construct and costained with β-tubulin antibody and MitoTracker. In this experiment, mitochondrial clumping was used as a marker of transfection. As seen in pictures J to L of Fig. 5 the clustering of mitochondria was not accompanied by either MT depolymerization or cell shrinking. To conclude, both experiments clearly show the importance of KBP for the distribution of mitochondria in cells.

### Mitochondria aggregation and the role of KBP

**Figure 6:**
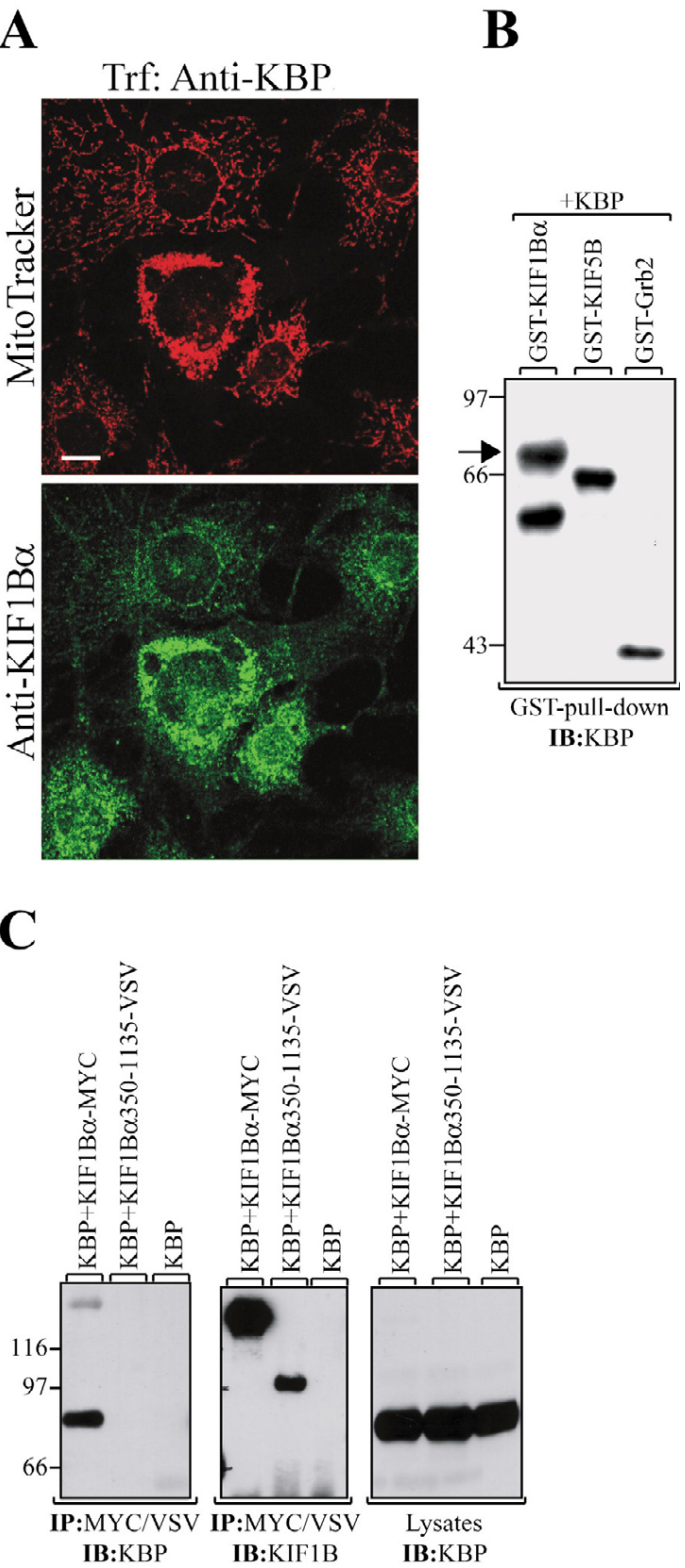
**KIF1Bα colocalizes with aggregated mitochondria**. **A**, NIH3T3 cells were transfected with anti-sense KBP and costained with MitoTracker (upper picture) and KIF1Bα polylonal antibody (lower picture). Bar, 10 μm. **B**, 1 μg of KIF1Bα and KIF5B GST-fusion proteins were bound to glutathione Sepharose and incubated with equal aliquots of the same lysate from KBP expressing 293 cells. As a control, GST-Grb2 fusion protein was used. The proteins were separated, transferred to nitrocellulose and immunoblotted with GST-KBP antiserum. Molecular mass markers are shown in kDa, and an arrow indicates the position of KBP. Trf, transfection. **C**, KBP was transiently overexpressed either alone or together with KIF1Bα-MYC or KIF1Bα350-1135-VSV. Proteins were immunoprecipitated with either VSV or MYC antibody and KBP was detected with the specific antibody. The filter was reprobed with KIF1B antibody, and KBP expression was confirmed as before.

The above results did not answer the question whether KBP is a mitochondrial receptor for KIF1Bα and is mediating the interaction KIF1Bα/mitochondria. We therefore analysed the localization of KIF1Bα in cells with a reduced KBP expression. As described before, cells were transfected with the anti-sense KBP construct and transfected cells identified by staining with MitoTracker. In addition, cells were stained with KIF1Bα antibody. As seen in Fig. 6A (top), a perinuclear localization of mitochondria indicates transfection with the anti-sense KBP construct. The bottom picture shows perinuclear localization of KIF1Bα and colocalization with aggregated mitochondria. This suggests that KIF1Bα is still associated with the mitochondria after down-regulation of KBP and that KBP does not function as an adapter.

In addition to KIF1B, conventional kinesin (KIF5B) also was described to mediate transport of mitochondria [21]. To confirm a specific role of KBP for KIF1Bα and not KIF5B, we tested the interaction between these proteins. Therefore, we constructed a plasmid encoding a GST fusion protein encoding amino acids 265–639 of KIF5B, corresponding to the previously used GST-KIF1Bα. Proteins were purified from bacteria and incubated with glutathione-sepharose and lysates from 293 cells transiently overexpressing KBP. As indicated in Fig. 6B, only GST-KIF1Bα associated with KBP. Neither GST-KIF5B nor GST-Grb2 (as a control) were able to bind KBP. The GST-fusion proteins are visible since the anti-KBP antiserum was generated using a GST-KBP fusion protein and indicate that similar amounts were used for the pull-down.

The above finding suggests that KBP does not bind the KIF1Bα's carboxyl-terminus, which was shown to mediate cargo interactions [16,22]. To identify the KBP-binding site on KIF1Bα we generated a mutant with an amino-terminal truncation of 349 amino acids (KIF1Bα350-1135-VSV) which was not able to bind KBP (Fig. 6C). Together with the data presented in Figure 6B this confines the binding of KBP to KIF1Bα between amino acids 270 and 350 at the carboxyl-terminus of the motor domain. Thus, KBP plays an important role in mitochondria distribution in cells but it is neither a mitochondrial receptor nor a general adapter protein for mitochondria transporting KLPs.

### KBP improves KIF1B motility

**Figure 7:**
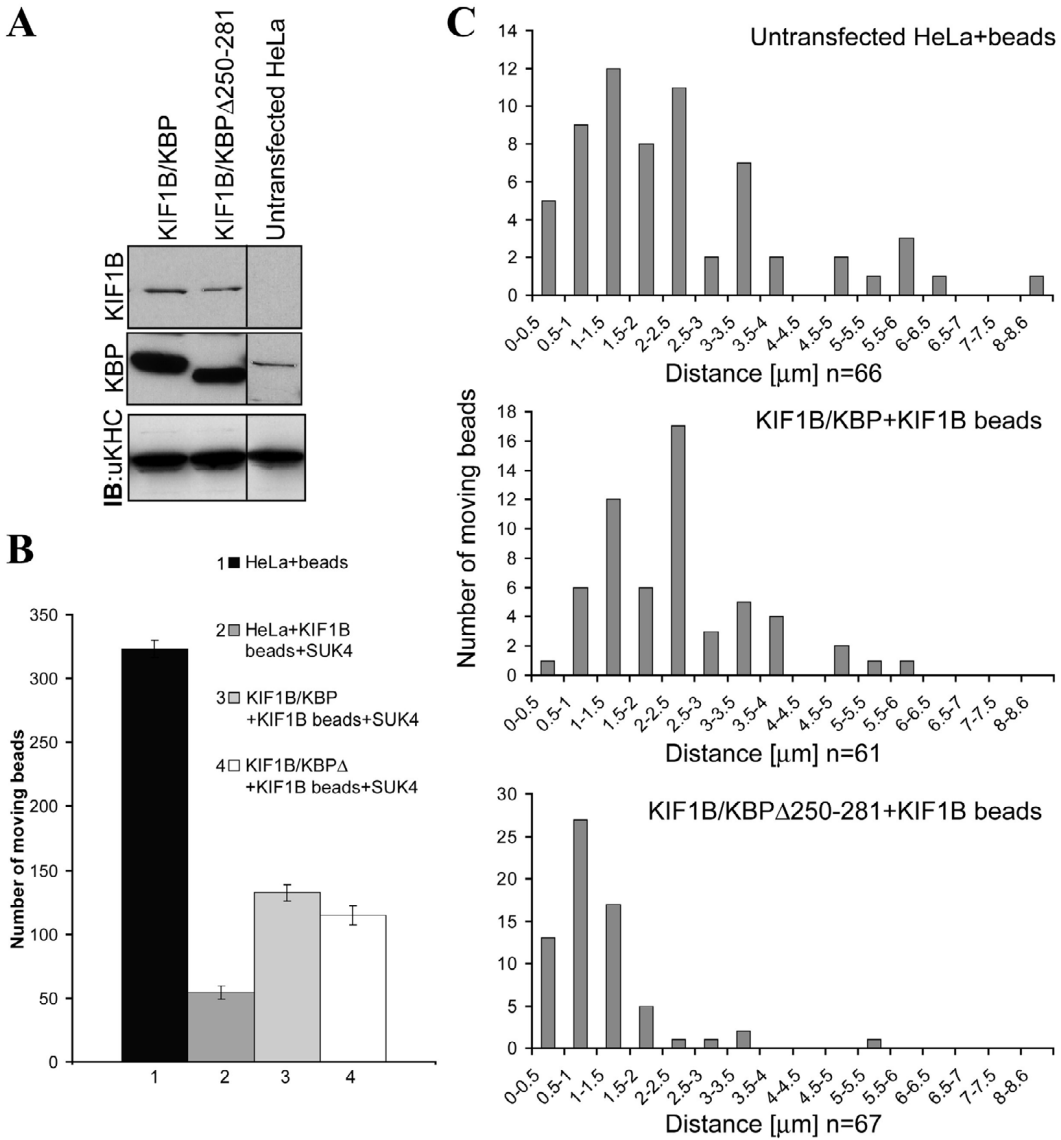
**KBP increases the motility of KIF1Bα**. **A**, extracts were prepared from HeLa cells that were either untransfected or transfected with KIF1Bα and KBP or KBPΔ251–281. The cells were cracked and cytosolic extracts separated by SDS-PAGE (20 μg of total protein) transferred onto nitrocellulose and blotted with KIF1B (top panel), KBP (midddle panel) and ubiquitous kinesin antibodies (bottom panel). **B**, uncoated or KIF1B antibody coated beads moving on MT sedimented on coverslips in flow cells were counted. Movements of beads were observed using VE-DIC in HeLa extracts as described in the Methods section. Each bar represents the number of beads counted during 5 minutes of observation on 10 planes in 3 independent experiments, where each plane was observed for 30 sec. **C**, distances traveled by beads in a particular extract were determined using RETRAC software. The total number of analysed beads (n) is indicated below each histogram.

KBP does not mediate the association of KIF1Bα with mitochondria since it is still found there when KBP is depleted. We therefore hypothesized that KBP could either increase the time of association of KIF1Bα with MT, or it could influence the motility of the KIF1Bα motor domain. To investigate this hypothesis we used latex beads in in vitro motility assays. Extracts were prepared from untransfected HeLa cells or from HeLa cells overexpressing KIF1Bα together with KBP or the KBP deletion mutant (Fig. 7A). Extracts from untransfected cells served as control for unspecific moving. The unspecific movements could be attributed to the large amount of kinesin heavy chain present in the extract (Fig. 7A), which bound beads. The KIF5B can be inhibited by SUK4 antibody [23], and indeed treatment of extracts with the antibody strongly reduced motility in HeLa extracts. Inhibition of unspecific movements could be further improved by coating the beads with KIF1B antibody. Both treatments together reduced the amount of moving beads by about 90% (Fig. 7B, bars 1,2). Upon overexpression of KIF1Bα together with KBP the number of moving beads was significantly higher than in extracts from untransfected cells. This number was not affected by additional co-overexpression of the KBP deletion mutant (Fig. 7B, bars 3 and 4). Therefore, we assume that more than 60% of the observed movements was caused by the overexpressed KIF1Bα. When analysing the velocities of moving beads, we noticed that the deletion mutant slightly decreased the velocity of the beads as compared with the wild type (Table 1). Most striking was the strong reduction of the distance traveled by beads in extracts coexpressing KBPΔ250–281, most of which was shorter than 1 μm, while in extracts coexpressing wild type KBP the beads traveled longer than 2 μm (Fig. 7C; for average values see Table 1). Taken together, our results indicate that KBP increases the motility of KIF1Bα.

**Table 1:** Summary of data from motility assays. Average values for velocities and distances traveled by beads are shown.

**Lysate**	**Max velocity**		**Distance traveled along MT**	
	**[μm/s]**	**St. error**	**[μm]**	**St. error**
KIF1B /KBP (n = 61)	0.82	0.022	2.32	0.15
KIF1B/KBPΔ250–281 (n = 67)	0.69	0.02	1.07	0.098
P-value	2.92E-05		1.25E-09	

## Discussion

In the present study we report on the identification of the new ***K***IF1 ***b***inding ***p***rotein KBP. The association of KBP with KIF1Bα was verified in vitro, in 293 cells transiently overexpressing both proteins and in untransfected BHK cells. Anti-sense experiments revealed that KBP is required for a proper distribution of mitochondria in the cell. Finally, we showed that KBP increases KIF1Bα motility in vitro.

The bait used in the yeast two-hybrid screen that led to the identification of KBP contained the sequence from the motor domain to the PTPD1 binding region (amino acids 261–800). Additional data from binding experiments of KBP to KIF1C and KIF1Bα deletion mutants allowed us to narrow down the binding sequence of KIF1B/C for KBP to the carboxyl-terminal region of the motor domain. A high amino acid sequence homology of 90% between KIF1C and KIF1Bα in this region supports the conclusion that KBP can bind to both proteins, and a manuscript describing the role of KBP for KIF1C is in preparation. It is important to note that the binding region of KBP is located close to the K-loop, a region in KIF1 KLPs that increases the affinity of the motor domain for MT [24]. For KIF1B, several splice variants involving or located next to the K-loop are known [13], and it will be interesting to check whether the presence of these variants has an effect on interaction with KBP [12]. An interaction with the KIF1Bβ variant would open a role for KBP as a player in neurodegenerative diseases. Recently, the role of the K-loop in a 679 amino acid construct of the rat orthologue of KIF1C was analysed [25]. In in vitro experiments this protein was not chemically processive, the K-loop increased the affinity for MT and stabilized a weak binding mode with ADP in the active site. The hypothesis was formed that rat KIF1C could work in teams of motor proteins, which could generate processive movement of attached organelles. This would be a similar mechanism as proposed for KIF1A [22]. In our motility assays we observed that KBP increased the motility of beads in extracts from KIF1Bα overexpressing cells. When the motor protein was overexpressed with KBP, it traveled average distances of 2–3 μm and maximally 6 μm, while with KBPΔ251–281 we observed KIF1Bα moving an average of 1 μm. These experiments do not contradict the data of Rogers et al. and Klopfenstein et al. since it is possible that KBP facilitates grouping of motor proteins and thus increases the motility. However, it also indicates an alternative mechanism where associated proteins like KBP could increase the motility of single molecules of KIF1B.

Numerous proteins have been identified that interact with KLPs [26,27]. These proteins may determine the localization of the KLP complex or which cargo is transported. Very little, however, is known about associating proteins that regulate either the cargo binding of the KLP or its activity. Such a KLP associating protein that is required for the KLP function has been described in yeast. The MT minus-end directed motor protein Kar3p is important for meiosis I in *S. cerevisiae*, and a knock out leads to an arrest in prophase I [28]. The associating protein Cik1p targets Kar3p to MT, implicating this as a reason why loss of Cik1p also impairs meiosis, although the effect is less severe [29]. Several studies describe a regulatory role of the carboxyl-terminus in conventional kinesin processivity. Kinesin light chains were reported to inhibit the binding of the heavy chain to MT [30]. Further, the kinesin tail domain by itself has an ATPase inhibiting role that is relieved by cargo binding [31-33]. One function of the light chains and the carboxyl-terminus of the heavy chain may thus be to ensure that kinesin is active only in the presence of its cargo. However, most of these studies have been done in vitro and cannot account for effects of regulating proteins.

The drastic consequences of the loss of KBP expression for mitochondria localization could also indicate that KBP is targeting KIF1Bα to the mitochondria. However, KIF1Bα is attached to mitochondria also after depletion of KBP (Fig. 6A). In addition, KLPs normally bind their cargo with carboxyl-terminal sequences, whereas KBP interacts with KIF1Bα amino-terminal sequences. Therefore, we do not think that KBP is important for the cargo – kinesin connection. Alternatively and mentioned before, KBP may regulate the KIF1Bα activity, i.e., in the absence of KBP, KIF1B is not or less able to move mitochondria.

Only two widely expressed KLPs have been reported to transport mitochondria, KIF1Bα and KIF5B, while KIF5A and KIF5C are expressed predominantly in neuronal tissues [34]. KIF5B did not interact with KBP, and it should not be affected by KBP depletion. Thus, even if there is some expression of KIF5B, a major role of KIF1Bα for mitochondria transport in NIH3T3 cells is likely. Recently, the *Drosophila *protein MILTON was described that associates with the KIF5 ortholog kinesin heavy chain and may play a role similar to KBP since its mutation impairs MT-plus end directed mitochondrial transport in axons and synapses [35]. In addition to KLPs, dynein motor proteins probably can transport mitochondria in cells [36], and this transport would occur in a MT minus-end direction. When force moving mitochondria to the MT plus-ends is reduced, a perinuclear localization of the mitochondria would result. On the other hand, a complete depolymerization of MTs by nocodazole treatment led to a diffusion of mitochondria throughout the cytoplasm [21], demonstrating the specificity of the consequences of KBP depletion.

## Conclusion

We have shown earlier that protein tyrosine phosphatase D1 and members of the 14-3-3 protein family interact with KIF1C, implying its regulation by phosphorylation. In this manuscript, we have identified the new protein KBP as a KLP interacting protein that is required for KLP activity. This suggests that vesicle transport in cells may be subject to regulation at the level of KLP – microtubule interaction and implicates KBP as a physiological mediator.

## Methods

### Yeast two-hybrid screen

Two fragments of the KIF1C cDNA encoding amino acids 435–622 (corresponding to the U104 domain) or amino acids 261–800 were cloned into the LexA fusion protein vector pBTM116 and transformed into the *Saccharomyces cerevisiae *strain L40 (MATa trp1 leu2 his3 LYS2::lexA-HIS3 URA3::lexA-lacZ [37]), generating the L40 LexA-KIF1C_U104 _and L40 LexA-KIF1C_298–840 _strains, respectively. A brain cDNA library fused to the GAL4 activation domain in the pACT2 vector (Clontech) was transformed into the L40 LexA-KIF1C_U104 _and L40 LexA-KIF1C_298–840 _strains and 5 × 10^6 ^transformants were screened for interaction as described [38]. Yeast plasmid DNA was isolated from His^+ ^β-Gal^+ ^colonies, rescued into *Escherichia coli *HB101, retransformed into L40 LexA-KIF1C_298–840_, and yeasts assayed for β-galactosidase activity and growth on medium with complete supplement lacking amino acids Trp, Leu, Ura, Lys, and His (Bio101). The specificity of the interaction between KIF1C_298–840 _fragment and potential candidates was proven by transforming the candidate plasmid also into a L40 LexA and a L40 LexA-laminin strain.

### DNA sequence analysis

Clones interacting specifically with KIF1C in the two-hybrid screen were subjected to sequencing by the dideoxynucleotide chain termination method. Non-redundant databases (GenBank, EMBL, DDBJ and PDB) were searched for homologous sequences using the BLAST program [39,40].

### Northern blot analysis

A multiple tissue Northern blot was obtained from Clontech. Hybridization was performed at 67°C with 5 × 10^6 ^cpm/ml of ^32^P-random primed DNA probe as described by the manufacturer.

### cDNA cloning and protein purification

For transient expression in 293 and NIH3T3 cells, all cDNAs were cloned by standard procedures into the cytomegalovirus immediate early promoter-based expression plasmid pRK5 [41]. To clone a KIF1Bα encoding cDNA, the 3'-part of the open reading frame was received from clones FH12320 (1941–3721 bp of this clone) and HJ02790 (1–685 bp of this clone) known in HUGE Protein Database as genes KIAA1448 [42] and KIAA0591 [43], respectively. The 5'-part was amplified in a PCR reaction using a human brain cDNA library as a template. A cDNA fragment encoding the amino acids 265–639 of the human version of KIF5B was amplified with specific primers in a similar way. The corresponding region of KIF1Bα (amino acids 270–641) was cut out from the KIF1Bα cDNA using restriction enzymes, and both fragments were cloned into a pGEX vector. The corresponding fusion proteins were produced in *Escherichia coli *BL21 (Novagen) or 298 F' strains and purified as described [44].

### Antisera

The KIF1B and KBP rabbit polyclonal antisera were raised against glutathione-S-transferase (GST) fusion proteins of KIF1B (amino acids 743–1153) and KBP (full length), respectively. Monoclonal antibodies used were derived from the following hybridoma: kinesin heavy chain – H2; phosphotyrosine – 4G10; β-tubulin – KMX1; vesicular stomatitis virus (VSV) epitope tag – p5d4; KIF5 – SUK4; hemagglutinin (HA) epitope tag – 12CA5.

### Cell lines and cell culture

293 cells were grown in F12/Dulbecco's modified Eagle's medium supplemented with 10% fetal calf serum and 2 mM glutamine. BHK, HeLa, NIH3T3 and C_2_C_12 _cells were maintained in Dulbecco's modified Eagle's medium supplemented with 10% fetal calf serum and 2 mM glutamine.

### Transient expression, cell lysis, and immunoprecipitation

Transfection of 293 cells and NIH3T3 cells was performed using the method of Chen and Okayama [45]. The cells were lysed in 1 ml of lysis buffer/10-cm plate (1% Triton X-100, 50 mM HEPES, pH 7.5, 10% glycerol, 150 mM NaCl, 1.5 mM EGTA, 10 mM sodium pyrophosphate, 100 mM NaF, 1 mM sodium orthovanadate, 10 μg/ml aprotinin, 1 mM phenylmethylsulfonylfluoride) and the lysates were precleared by centrifugation at 13,000 *g *for 15 minutes at 4°C. The lysates were adjusted for equal protein concentration, the appropriate antibody (3 μl of serum or 2 μg of purified antibody) and either Protein A- or Protein G-Sepharose were added, and the lysates were incubated for at least 3 hours at 4°C on a turning wheel. The immunoprecipitates were washed with HNTG buffer (20 mM HEPES, pH 7.5, 150 mM NaCl, 0.1% Triton X-100, 10% glycerol, 10 mM NaF, 1 mM sodium orthovanadate), separated on an 8 or 10% SDS-polyacrylamide gel, transferred to a nitrocellulose membrane, and incubated with the appropriate primary antibody. After three washes, the membranes were incubated with secondary horseradish peroxidase-conjugated goat anti-mouse or goat anti-rabbit antibodies (Sigma). After three washes they were visualized using the ECL system. Before reprobing, blots were incubated for 30 minutes in 62.5 mM Tris-HCl, pH 6.8, 2% SDS, and 0.1% β-mercaptoethanol at 55°C.

### In vitro binding assays

For in vitro binding assays, 293 cells transiently overexpressing KBP were lysed and incubated for 4 hours at 4°C with GST or GST-KIF1Bα/KIF5B fusion proteins immobilized on glutathione-Sepharose. After washing with HNTG buffer, the proteins were separated by SDS-PAGE and analysed by Western blotting.

### Immunofluorescence

NIH3T3 cells were grown on uncoated glass coverslips, fixed for 20 minutes in ice-cold methanol at -20°C, washed with phosphate-buffered saline and incubated for 10 minutes with 0.1% NaBH_4 _and 0.1 M glycine in phosphate-buffered saline to block autofluorescence. Nonspecific antibody binding was blocked for 45 minutes with PBS with 0.045% fish gelatin containing 5% normal goat serum and 1% bovine serum albumine. Incubation with primary antibody was done for 1.5 hours at 37°C after dilution in PBS/gelatin containing 5% normal goat serum. The affinity purified KBP and KIF1Bα antibodies were used at concentrations of 10 ng/μl and 20 ng/μl, respectively. The β-tubulin antibodies were diluted as recommended by the manufacturer, and the monoclonal VSVantibodies at a concentrations of 20 ng/μl. After five washes with PBS/gelatin, primary antibody binding was detected with isotype-specific secondary antibody conjugated with either DTAF (goat anti-rabbit, Dianova) or Alexa (Alexa Fluor 546 goat anti-mouse and goat anti-rabbit and Alexa Fluor 488 goat anti-mouse, Molecular Probes). The coverslips were mounted in PermaFluor (Immunotech). To allow unbound protein to diffuse out, extraction of cells with 0.5% Triton X-100 was performed prior to fixation for 5–10 seconds [46,47]. MitoTracker (Molecular Probes) staining was done according to manufacturer's recommendations.

Pictures were taken with a Leica TCS NT/SP Laser Scanning Confocal Microscope supplied with HCX PL APO immersion oil objective of magnification 63× and aperture 1.32–0.62. The pictures were prepared using TCS software supplied by the manufacturer.

### Cell extracts and motility assays

Cell extracts were prepared in BRB80 buffer (80 mM Pipes, pH 6.8, 2 mM MgCl_2_, 1 mM EGTA) supplemented with protease inhibitors, through multiple passing through cell cracker (HGM, Heidelberg, Germany). The nuclei were spun down and the supernatant was spun again at 120,000 g to separate cytosol from membrane fraction The concentration of the cytosolic fraction was adjusted to 1 mg/ml and supplemented with 1 mM DTT, 20 μM taxol, 2 mg/ml BSA, 5 mg/ml casein and 1/20 volume energy mix (150 mM creatine phosphate, 20 mM ATP, 20 mM MgCl_2_, 2 mM EGTA, pH 7.7).

For motility assays with latex beads, 1 μl of beads (2.5% solid-latex, carboxylate 0.1 micron microspheres; Polysciences, Inc., Warrington) was left untreated or incubated for at least 30 minutes with 4 μl of KIF1B antibody solution (3 mg/ml). Then the antibody/beads suspension was diluted 1:6 in BRB80, 1 mM DTT, 20 μM taxol, 2 mg/ml BSA, 5 mg/ml casein and 1/20 volume energy mix. One μl of beads was mixed with 9 μl of cell extract and floated into a flow cell with coverslips coated with microtubules. The beads were left for 5 – 7 minutes in a humid chamber to sediment onto the microtubules. To inhibit unspecific KIF5B driven movements, SUK4 antibody was incubated with extracts for 10 minutes at a final concentration of 0.25 mg/ml, and then the extracts were used in the assay. Flow cells were prepared as described earlier [48], and microtubules were isolated from pig brain as described [49].

Motility was observed using video-enhanced differential interference contrast microscopy (VE-DIC) in real time (Olympus optics BX60 microscope, equipped with DIC optics; Japan, Tokyo). To determine the rates and distances, RETRAC software (Dr. N. Carter, Marie Curie Research Institute, Oxted, Surrey, UK) was used.

## List of abbreviations

ER: endoplasmic reticulum

GST: gluthatione-S-transferase

HA: hemagglutinin

KBP: KIF1-binding protein

KLP: kinesin-like protein

MT: microtubules

VSV: vesicular stomatitis virus

## Authors' contributions

MJW performed most experiments and contributed to the manuscript, MM did the yeast-2-hybrid screens, CD contributed plasmid constructs and antibodies, HUH participated in the study design, and RL conceived the study, contributed to the experiments and wrote the manuscript. All authors read and approved the final manuscript.
